# Developmental mechanisms directing early anterior forebrain specification in vertebrates

**DOI:** 10.1007/s00018-013-1269-5

**Published:** 2013-02-09

**Authors:** Cynthia Lilian Andoniadou, Juan Pedro Martinez-Barbera

**Affiliations:** Birth Defects Research Centre, UCL Institute of Child Health, 30 Guilford Street, London, WC1N 1EH UK

**Keywords:** Anterior forebrain, Neural induction, AVE, Hesx1, Six3, Tcf3

## Abstract

Research from the last 15 years has provided a working model for how the anterior forebrain is induced and specified during the early stages of embryogenesis. This model relies on three basic processes: (1) induction of the neural plate from naive ectoderm requires the inhibition of BMP/TGFβ signaling; (2) induced neural tissue initially acquires an anterior identity (i.e., anterior forebrain); (3) maintenance and expansion of the anterior forebrain depends on the antagonism of posteriorizing signals that would otherwise transform this tissue into posterior neural fates. In this review, we present a historical perspective examining some of the significant experiments that have helped to delineate this molecular model. In addition, we discuss the function of the relevant tissues that act prior to and during gastrulation to ensure proper anterior forebrain formation. Finally, we elaborate data, mainly obtained from the analyses of mouse mutants, supporting a role for transcriptional repressors in the regulation of cell competence within the anterior forebrain. The aim of this review is to provide the reader with a general overview of the signals as well as the signaling centers that control the development of the anterior neural plate.

## Introduction

The overall anatomical structure of the mature vertebrate brain has been highly preserved throughout evolution, although the degree of development of particular domains can vary enormously between species. From fish to mammals, the brain can be divided into three main areas according to their position along the antero-posterior axis: the fore-, mid-, and hindbrain. The anterior forebrain includes the telencephalon (cortex, hippocampus, and basal ganglia), hypothalamus, prethalamus as well as the eyes and visual tracts (optic chiasm). The posterior forebrain comprises the thalamus, epithalamus, and pretectum [59, 128, 129]. The entire brain has a common origin during early embryonic development in a single-layered pseudostratified epithelium called the neural plate (NP). The NP becomes molecularly distinct to surface ectoderm (destined to form the skin) soon after gastrulation when neural induction takes place. Subsequently, it is divided into domains along the antero-posterior and medio-lateral axes characterized by specific gene expression patterns, which will eventually give rise to brain structures at later stages of development [121, 130]. Using embryological techniques such as single cell labeling and cell transplantations or genetic approaches (i.e., genetic tracing using mouse lines expressing Cre recombinase), the origins of the distinct components of the mature brain have been mapped in the early NP [25, 27–29, 63, 123, 159]. Such studies have also demonstrated a remarkable degree of conservation between species at early embryonic stages, despite the considerable differences in the shape and size of their mature brain structures.

The focus of this article is to revise the major findings that have contributed in the generation of a model of how the forebrain is formed during the early stages of development and in particular, the mechanisms underlying anterior forebrain formation. We have attempted to provide a historical perspective of the milestones that provided the foundations to this model and to link these with more recent discoveries. We aim to discuss findings obtained from studies in different animal models, often first describing the results obtained from the initial experiments regardless of the species and commenting on the degree of conservation in other experimental models. We will discuss the relevance of essential signaling centers as well as critical signals involved in the process of anterior forebrain formation, paying special attention to the WNT/β-catenin pathway. To learn about the numerous intrinsic factors that are also essential for normal anterior forebrain development we refer the readers to other published review articles [9, 59, 76].

## Neural induction and antero-posterior axis formation: a historical perspective

**Fig. 1:**
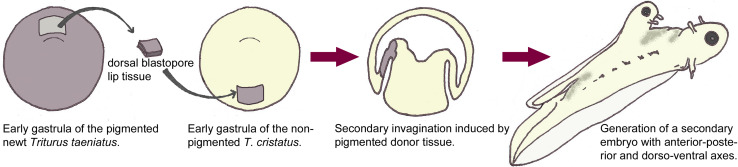
Dorsal blastopore lip tissue of the early gastrula displays organizing activity. Excision of dorsal blastopore lip tissue from the pigmented newt *Triturus taeniatus* and transplantation in the region of presumptive ventral epidermis in a non-pigmented *Triturus cristatus* early gastrula. The donor tissue induces a secondary invagination in the host embryo and exerts organizing activity to surrounding host tissues. A secondary embryo forms where the donor tissue contributes mainly to notochord and prechordal mesoderm structures and re-organizes the normally ventral surrounding host tissues, which are dorsalized into neural tube and mesoderm (adapted from Gilbert, Developmental Biology)

The study of anterior NP (ANP) specification, neural induction, and antero-posterior (AP) axis formation are intimately linked. Experiments by Hans Spemann and Hilde Mangold [134] demonstrated that an entire ectopic axis extending from the forebrain to the spinal cord could be induced in a host newt embryo by a group of cells from the dorsal blastopore lip (Fig. 1). This ectopic axis was complete in that a neural tube encompassing forebrain (i.e., ectopic eyes) and spinal cord had developed over an ectopic notochord and was flanked by somites. Newt species with white or dark pigments were used to discriminate the contribution of the host and the donor tissues to the ectopic axis. The ectopic neural axis was comprised of mostly host cells, demonstrating that the donor cells have inducing properties and was named “the organizer”. Moreover, the abilities of the organizer were stage dependent, as dorsal blastopore lip cells from younger embryos were capable of inducing an entire ectopic neural axis (brain and spinal cord), but older ones gave rise to ectopic axes lacking the eyes. This gave support to a model whereby the activities of the organizer can be divided into the “head” and “trunk” organizers, with early activities inducing anterior neural identity and late activities only posterior neural fates. This concept, however, was challenged many years later by another developmental biologist, Pieter Nieuwkoop [106], who carried out an ingenious transplantation experiment using the urodele embryo. He inserted pieces of naive ectoderm along the rostro-caudal neural axis of the host embryo and later analyzed the presence of anterior (forebrain) or posterior neural structures. He observed that forebrain structures developed at the distal tip of the flap and posterior structures at the base, matching the neural identity of the level at which it was initially inserted in the host. This led him to postulate the “induction-transformation” model, whereby neural tissue is initially induced with anterior character and subsequently caudalized by signals from the organizer with transforming activity.

Some basic principles derived from these experiments have survived to date. Structures with similar activities to the amphibian organizer were identified in other vertebrate species including chick (Hensen’s node), zebrafish (embryonic shield), and mouse (node). In these, the transplantation of the organizer led to similar results to those in amphibians [124, 138, 153]. However, it came as a surprise that transplantation of the mouse node gave rise to an incomplete axis lacking the anterior forebrain [10, 145]. The possibility existed that the difficulty of such transplantations in the small mouse embryo may affect the results and so, the inability of inducing a full axis may be a “technical flaw”. Even in *Xenopus*, where these experiments are easier due to the larger embryo size, only a small proportion develop a full neural axis upon dorsal blastopore lip transplantation. An alternative explanation may be that the transplanting node only contained the posterior inducing activity, as does the late or trunk organizer of the amphibian embryo. However, even the transplantation of cells from the “tip of the primitive streak” at early streak stages (early gastrula organizer, EGO), which contains precursors of the node, failed to induce a full axis [145].

**Fig. 2:**
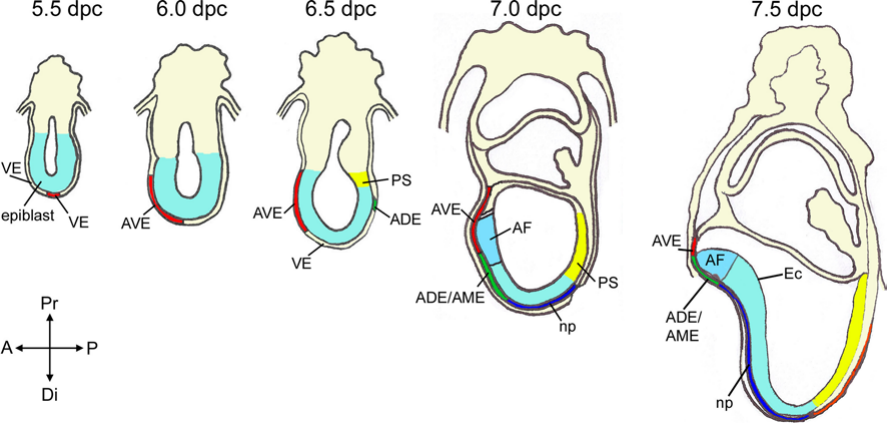
Specification of tissues involved in patterning the anterior forebrain in the post-implantation mouse embryo and establishment of the anterio-posterior axis. *AVE* cells are induced to form by signals from the epiblast acting on *VE* at the distal tip of the embryo by 5.5 dpc. *AVE* cells migrate anteriorly at 5.5 dpc, reach the boundary between the epiblast and extra-embryonic ectoderm in approximately 5–6 h and then start to move laterally. At 6.5 dpc, the *PS* elongates and the anterior movements of the *ADE/AME* displace and intermingle with the AVE. The forebrain domain including future anterior and posterior forebrain is patterned within the anterior portion of the presumptive neural ectoderm that overlies AVE and ADE/AME tissues. By 7.5 dpc, the prospective anterior forebrain neural ectoderm overlies the ADE/AME and posterior neural tissue is underlied by the notochordal plate. *VE* visceral endoderm, *AVE* anterior visceral endoderm, *ADE* anterior definitive endoderm, *AME* anterior mesendoderm, *np* notochordal plate, *PS* primitive streak, *AF* anterior forebrain primordium, *Ec* neural ectoderm

The answer to this discrepancy observed in the mouse was simpler: the node/EGO did not contain all the information required to induce an entire neural axis because the anterior visceral endoderm (AVE) was necessary for anterior neural tissue to develop (Fig. 2) [146]. There have been numerous reviews on the subject, so we will just summarize some major findings that have provided support to this idea [11, 12, 94, 135, 143]. The AVE was found to contain a group of cells, which express several genes relevant for head formation (i.e., *Otx2*, *Lhx1*, *Foxa2, Cer1*, *Lefty1*, and *Dkk1* among others) and was patterned prior to any signs of primitive streak formation. Moreover, genetic evidence demonstrated the requirement of several of these genes specifically in the AVE for normal antero-posterior axis formation and anterior forebrain development [1, 38, 70, 71, 101, 114, 117, 127, 151, 161]. This was in addition to a critical role for some of these genes within the anterior neural plate (e.g., *Otx2*) [1, 17, 117]. Tissues equivalent to the mouse AVE, according to their location and molecular expression patterns, exist in embryos of chick (hypoblast), zebrafish (dorsal syncytial layer), and *Xenopus* (yolky cells of the vegetal pole), suggesting a conserved role in the vertebrate embryo [58, 65, 136]. Currently, it is broadly accepted that the AVE plays a critical role in the establishment of the AP axis and during anterior neural plate induction [86, 118, 157]. However, the formation of the anterior neural plate does not exclusively rely on AVE activities and there must be cooperation with other signaling centers such as the gastrula organizers and axial mesendoderm underlying the developing anterior neural plate. As proposed by Nieuwkoop, the synergism of these signaling centers aims to protect the anterior region of the epiblast from posteriorizing signals, which would otherwise bestow caudal neural character (i.e., any neural tissue that is not anterior forebrain). Therefore, neural tissue develops anterior character unless exposed to caudalizing signals. His induction-transformation model appears to match normal neural patterning more closely than the head-trunk organizer hypothesis.

## The influence of extrinsic signals in anterior neural induction

It is broadly accepted that anterior neural induction requires the inhibition of the BMP, TGFβ, and WNT/β-catenin pathways. This model has been built from a bulk of evidence based on gene expression, loss- and gain-of-function experiments, chimeric analyses, and embryological data in *Xenopus*, zebrafish, chick, and mouse.

Historically, experiments in *Xenopus* had a major impact on the origin of this model. Animal caps that are cultured as intact explants become surface ectoderm, but if cells are disaggregated, hence released of extrinsic cues, they become neural ectoderm of anterior identity [55]. This has been one of the pillars sustaining the “default” model of neural induction, which suggests that at pre-gastrula stages, the default fate of naive ectoderm is usually neural (i.e., brain) rather than surface (i.e., skin) ectoderm [86]. In fact, treatment of dispersed animal cap cells with BMPs could convert them into epidermis [155]. These initial findings have been confirmed in different experimental models, including zebrafish [15, 44] and mice. Mouse embryos deficient for the BMP receptor 1A (*Bmpr1a* ^−*/*−^), the main receptor for BMP2 and 4, exhibit premature neural induction at pre-gastrula stages with expression of anterior markers *Hesx1* and* Six3* [35]. This is a similar phenotype to that observed in *Nodal* ^−*/*−^ mutants, and indeed *Bmpr1a* ^−*/*−^ embryos show no expression of Nodal [21, 35]. These findings fit well with the concept that inhibition of BMP/TGFβ signaling is a prerequisite for neural induction. BMP inhibitors such as noggin and chordin are expressed in the vertebrate organizers and play a role in neural induction by preventing BMP signaling [6, 118, 162]. This is beautifully exemplified in *Xenopus*, where the simultaneous depletion of noggin, chordin, and follistatin function by means of specific morpholinos, prevents the acquisition of neural fates demonstrating the critical role of BMP signaling inhibition during normal neural induction [66]. In addition, this research shows that there is a significant degree of functional redundancy, which may complicate the interpretation of data obtained in different animal models when addressing the requirement of specific signaling pathways [105].

Despite the robust evidence supporting the inhibition of BMP signaling for neural induction to occur, it was shown that this is not sufficient to induce an entire ectopic neural axis containing anterior and posterior components. The ability of Cerberus, a secreted inhibitor of nodal, BMP, and WNT molecules to induce an ectopic full axis when over-expressed in *Xenopus* confirmed the necessity to repress the BMP/TGFβ and WNT pathways for head formation to occur [16, 51, 64, 115]. WNT molecules were found to exert caudalizing activity on neural tissue. For instance, treatment of chick ectodermal explants fated to become anterior forebrain could be transformed into posterior neural tissue by exposure to WNT molecules [108] and *Wnt8c* over-expression in mouse embryos caused an expansion of midbrain markers at the expense of anterior forebrain [116]. These data suggest that once neural tissue has been induced, it must be protected from caudalizing activities that would bestow posterior identity, as proposed by Nieuwkoop.

Other molecules, such as retinoic acid (RA), were also shown to act as caudalizing factors, by promoting posterior fates in the neural plate [39]. In vivo, retinoic acid is synthesized from vitamin A (retinol) by the action of retinol dehydrogenases, which are expressed in the posterior regions of the embryo, but not in the anterior neural plate at the time of neural induction. In contrast, enzymes such as CYP26, a cytochrome P450 enzyme that degrades RA, is expressed at high levels in the anterior neural plate [56, 78]. This differential expression of synthesis and degradation enzymes is thought to create a RA signaling gradient along the AP axis (high-anterior and low-posterior) [90]. Fibroblast growth factors (FGFs) have also been shown to have caudalizing action on neural tissue in chick and *Xenopus* [97] and in mouse, exposure of prospective anterior forebrain tissue to FGF4 resulted in lack of expression of anterior forebrain markers and expansion of posterior markers [33].

FGF signaling has also been involved in the initiation of neural induction. Activation of FGF signaling has been proposed to be required prior to or independent to the inhibition of BMP signaling [43, 77, 88, 140]. Likewise, WNT signals have also been proposed to be required for neural induction in chick embryos [7, 112, 156]. However, there is also evidence suggesting that FGF signals are dispensable for the process of neural induction [154]. FGF signaling plays multiple and often contrasting roles in the early embryo as well as in pluripotent stem cells, and conclusions may vary depending on the particular experimental conditions and the assays used [81, 152]. Reconciling, at least partially, these discrepancies, elegant experiments have led to propose a model by which the activation of the FGF and WNT signaling pathways inhibits BMP signaling by promoting phosphorylation of Smad1 in a region of the molecule leading to its degradation and reduced nuclear localization [7, 40, 48, 113]. These findings are in line with experiments showing that inhibition of Smad1 and Smad2 is sufficient to induce neural character [23]. Together, these studies suggest that the activation of both the FGF and WNT pathways converge with the inhibition of BMP signaling at the levels of Smad1 and reinforces the idea that BMP signaling inhibition may be a conserved requirement for neural induction. They also suggest that there may be some species-specific differences regarding the temporal requirement of FGF signaling for neural induction. It will be important in the future to delineate the role of these specific pathways (e.g., retinoic acid, TGFβ, BMP, FGF, and WNT) and we anticipate that in vitro neural differentiation experiments using mouse embryonic and epiblast stem cells may provide important insights [26, 41, 54, 84, 102, 104, 131, 137, 142].

## Signals from both the AVE and gastrula organizers are required for normal anterior neural induction

The experiments described above led to a working model and identified major molecular players deployed during anterior neural induction. It is now clear that there is an involvement of at least two signaling centers for normal anterior neural induction, the AVE and the gastrula organizers. Relevant questions to further comprehend anterior neural induction are when during normal development, is the inhibition of the BMP/TGFβ and WNT pathways required? And which tissues do signals responsible for their inhibition derive from?

The visceral endoderm (VE) derives from a few cells lining the inner cell mass and the blastocoel cavity of the implanting 4.5 dpc mouse embryo (Fig. 2). At 5.0 dpc, VE is a single cell epithelial sheet surrounding the naive epiblast, fated to give rise to the embryo proper and the extraembryonic ectoderm. At this stage, the precursors of the AVE are molecularly identifiable at the distal tip of the conceptus (distal visceral endoderm, DVE) by the expression of genes such as *Hex*, *Lefty1* (a secreted inhibitor of Nodal) and *Cer1* (an inhibitor of Nodal as well as WNT and BMP molecules) [11]. They are induced at the distal tip of the egg cylinder through the co-operative inductive action of the Nodal and MAPK signaling pathways, whose action is restricted to the distal tip by repressive signals from the extra-embryonic ectoderm [19, 24, 119]. Recent elegant lineage tracing experiments have demonstrated that the AVE is derived from specific primitive endoderm cells of the pre-implantation blastocyst [144]. At the onset of gastrulation (6.5 dpc in the mouse), the AVE is located in the antero-proximal region of the embryo underneath the embryonic/extraembryonic border. This final location of the AVE requires the antero-proximal movement of the DVE cells by cell intercalation into the VE cell layer [149, 150]. Therefore, it is clear that prior to the onset of gastrulation, the AVE has had ample opportunity to affect the patterning of the overlying anterior epiblast. However, it seems unlikely that the AVE can induce permanent neural character on its own; mouse embryos deficient for either *Wnt3* or *Ctnnb1* (encoding β-catenin), express AVE markers, but there is no activation of neural markers in the epiblast and therefore, no neural induction [62, 89, 94]. Corroborating these genetic data, ectopic transplantation of chick hypoblast can induce naïve ectoderm to express transient expression of neural markers, suggesting a priming effect during neural induction, but the stabilization of neural character requires BMP inhibitors and/or organizer activities [2].

Cell intermingling in the epiblast of the pregastrula mouse embryo is very high [49] and fate map analyses have shown that the most antero-proximal region of the epiblast overlying the AVE at these stages is not fated to give rise to the anterior neural plate. Rather, the prospective neural plate precursors are located in the antero-distal region of the embryo [85]. It is likely that the function of the AVE at these pre-gastrula stages is not only to “prime” the epiblast for anterior neural induction, but also to position the primitive streak, hence for the establishment of the AP axis. Genetic evidence from mouse mutants supports this idea. For example, embryos lacking *Cer1* and *Lefty1* show abnormal AVE patterning concomitant with the formation of multiple ectopic primitive streaks [114]. In addition, embryological studies have shown that removal of the chick hypoblast also leads to the appearance of multiple primitive streaks [14].

In the mouse, at 6.5 dpc the PS forms in the posterior proximal region of the epiblast and will elongate during gastrulation so that by 7.5 dpc it reaches the distal tip of the embryo, where the node will form. Neural induction is thought to take place in the early/mid-streak stage embryo, when *Otx2* expression starts to be restricted to the anterior region of the epiblast. A few hours later, expression of anterior neural markers such as *Hesx1* and *Six3* becomes evident. The inducing activities of the tip of the PS and the gastrula organizers have been extensively studied in several species, including the mouse, and the evidence strongly suggests that the AVE and the gastrula organizers act together to induce anterior neural character. For instance, the EGO cannot induce anterior forebrain marker expression when transplanted to the lateral side of a host mouse embryo, but does so when combined with the AVE [145]. In agreement with the need for signaling activity from the AVE, the *Otx2* ^−*/*−^, *Foxa2* ^−*/*−^, and *Lhx1* ^−*/*−^ mutants have a molecularly identifiable EGO, but the AVE is not specified, resulting in an absence of anterior neural plate development [1, 5, 73, 117, 126]. However, the EGO activity is also required; as previously discussed, mouse embryos deficient for either *Wnt3* or *Ctnnb1*, express AVE markers at pre-gastrula stages, but do not form a PS and so do not express the molecular activities typical of the gastrula organizers, resulting in failure to undergo neural induction [62, 89]. The EGO/node and its derivatives express many common genes, among them inhibitors of the TGFβ and WNT pathways such as *Cer1*, *Dkk1*, *Nog* (noggin), and *Chrd* (chordin), reinforcing the idea that they act synergistically during anterior neural induction. Of note, the activities required for anterior neural induction may still be present in the absence of discernible anatomical structures. This is exemplified in the *Cripto* ^−*/*−^ mutants, which fail to gastrulate and so, neither the primitive streak or gastrula organizer are formed. However, genes normally expressed in the primitive streak and organizers are detected in the proximal region of the embryo. The AVE is also specified and typical AVE marker expression is observed at the distal tip of the mutant embryo. Despite this atypical arrangement of the AVE and organizer activities, anterior neural induction and patterning of the neural plate takes place in these mutants, although this is along the proximo-distal rather than AP axis [36].

Some experiments, however, have shown that the AVE and the mid-gastrula organizer (MGO, located at the distal tip of the primitive streak at mid-streak stage) can in isolation induce neural character. For instance, rabbit AVE is able to induce anterior neural character to naïve avian epiblast [74] and conversely, the MGO also appears to induce neural tissue in the absence of AVE [72]. There are several possible explanations for these findings, one being that the AVE or MGO may have the potential to induce anterior neural character when the experimental conditions are permissive. However, during normal development of the embryo both signaling centers are required. This is conceptually similar to the differences between regenerative and physiological potential of somatic stem cells when assessed in either transplantations or in lineage tracing experiments [132].

## Axial mesendoderm (AME) as a signaling center that maintains anterior neural character

Once the anterior neural plate has been induced, evidenced by the expression of anterior neural markers such as *Hesx1* and *Six3* at early neural fold stages, it is important to ensure the maintenance of this character and prevent its posteriorization. As with AVE at earlier stages, anterior axial mesendoderm (AME) tissue underlying the neural plate, plays a critical role in performing this function. The AME is a mixed population comprised of (1) anterior definitive endoderm (ADE), which is fated to give rise to the anterior foregut and its derivatives (e.g., liver, stomach, pancreas) [147]; (2) prechordal plate, which refers to endodermal and mesenchymal cells underlying the anterior region of the neural plate, which will form the anterior hypothalamus, telencephalon, and eyes; (3) notochordal plate, which gives rise to the notochord, underlying the developing neural plate caudal to the anterior hypothalamus (Fig. 2). The term AME in mouse is usually used to refer to the ADE and mesodermal component of the prechordal plate underlying the anterior neural plate. The origin of the AME is the gastrula organizers and the node. From the tip of the PS and subsequently from the node, a population of ADE cells expressing *Cer1* and *Hex* move anteriorly within the VE to underlie the rostral neural epithelium [13, 147]. Notochordal plate cells derive from the node and will follow the ADE and prechordal plate, but its location is more restricted to the midline of the neural plate. Of note, within the mouse ADE, endoderm cells from the prechordal plate and notochordal plate initially move within the existing layer of VE and not underneath, therefore they need to intercalate with VE cells in their migration [141]. This causes part of the VE to become displaced into the extraembryonic region, mainly in the anterior portion of the embryo, but VE cells also become trapped and contribute to the endodermal lining of the adult gut [79]. The notochord will form a rod before somitogenesis and will be situated between the endoderm and neuroectoderm as in other vertebrates.

The inducing activities of the anterior AME are well established [20]. Explant experiments have shown the capacity of these cells to induce and/or maintain anterior character (i.e., *Otx2* expression) in the vertebrate embryo [45]. In chick, the specific ablation of the ADE results in forebrain abnormalities with lack of telencephalic and eye development [158]. Likewise, absence of *Hex*, a transcription factor expressed in the ADE as it is formed from the tip of the PS, leads to patterning defects of the ADE and subsequent loss of anterior character (i.e., *Hesx1* and *Six3* expression) in the initially induced anterior neural plate [93]. The transcription factor *Lhx1*, and its co-factors *Ssdp1* and *Ldb1* are also necessary for normal brain development [42, 107, 126] and chimeric analyses have demonstrated its requirement in both the AVE and the AME [127]. The molecular activity of the anterior AME is thought to be similar to those of the AVE and gastrula organizers: to secrete inhibitors of the BMP/TGFβ and WNT pathways, hence protecting the anterior neural plate from posteriorizing signals. Supporting this notion, the AME expresses *Dkk1*, *Cer1*, *Nog*, and *Chrd* [34, 103, 163] and lack of these inhibitors, either single or in combination, is associated with defects in anterior AME and anterior forebrain specification [6, 34, 103, 162].

The role of the AME in protecting the ANP from caudalizing signals is also important for the establishment of other signaling centers within the neural plate responsible for the expansion and further patterning of forebrain tissue. For further detail on the relevance of these local signaling centers within the ANP, we refer the reader to other reviews [96, 122, 157]. One such center was initially discovered from elegant experiments in zebrafish, whereby the removal of the most anterior neural plate cells (anterior neural border, ANB) caused severe forebrain truncations [61]. The relevance for the ANB was also demonstrated in mouse embryos by means of ablation experiments where FGF8 was found to act as an essential signal for normal development of the anterior forebrain [130], which was further confirmed in several genetic studies [100, 110, 111, 139]. In the *Hex* ^−*/*−^ mutants, which show severe forebrain defects including lack of the telencephalon and eyes, *Fgf8* expression in the ANB is reduced or absent [93], and this appears to be caused by primary defects in ADE patterning. Corroborating this notion, removal of anterior definitive endoderm in chick leads to reduced *Fgf8* expression in the ANB and forebrain, including eye defects [158]. Neural crest cells have been shown to play a critical role in the maintenance of *Fgf8* expression in the ANR and expansion of the forebrain region [30, 31]. Another important signaling center in the forebrain is the zona limitants intrathalamica (zli), which is required for diencephalic development and is delineated by the expression of *Shh* [67, 82, 125]. It develops within the neural tube, specifically between the prethalamus and thalamus (ventral and dorsal thalamus, respectively), but its position coincides with the boundary between the prechordal and notochordal plates [96, 122], suggesting that underlying tissues may also play a role in its formation.

In summary, the induction of the anterior neural plate and specification of the AP axis require the continued inhibition of signals that would bestow the naïve epiblast of the pregastrula embryo with a non-neural or caudal neural character. AVE, gastrula organizers and anterior AME are sources of secreted inhibitors that ensure the protection of a population of epiblast cells from these signals. In addition, the AME is required for the proper positioning of local signaling centers within the anterior neural plate that are responsible for further growth and development of the area.

## Intrinsic regulation of cell competence: an additional level of protection of anterior neural character

In the previous section, we summarized a great deal of research pointing at the requirement for the anterior neural plate to be protected from signals that would transform it into posterior neural tissue. The activities of the AVE and gastrula organizers grant the epiblast anterior neural character, while the anterior AME acts to prevent its posterior transformation and refined patterning. This is achieved by the synergistic action of several secreted inhibitors. However, there are additional levels of protection within the neural plate itself, which prevent a posterior transformation of the ANP at stages when diffusion of secreted inhibitors from underlying tissues may be unable to reach this expanding region of the developing embryo.

Among the studied posteriorizing signals, it is the WNT/β-catenin pathway that appears to be critical in preventing anterior neural character. In *Xenopus* embryos there is a high-caudal to low-rostral gradient of activated WNT pathway, consistent with observations in other vertebrates [68]. For instance, in transgenic mouse lines expressing *lacZ* under the control of TCF/LEF binding sites, it has been shown that the anterior neural plate is initially devoid of X-gal staining, demonstrating that the WNT/β-catenin pathway is not activated in anterior neural precursors [4, 92]. Likewise, expression of the WNT/β-catenin target genes *Axin2* and *Sp5* is detected in posterior regions but not in the anterior-most part of the neural plate [3]. The necessity of the suppression of this pathway has been further analyzed in vitro and in vivo. As previously discussed, WNT molecules can transform cells initially fated to be anterior to acquire a caudal neural character [108]. In mouse embryos, genetic studies have demonstrated the need to negatively modulate the levels of WNT signaling for normal head formation [46, 47, 87, 116]. In zebrafish, mutations resulting in over-activation of the WNT pathway lead to posterior transformation of telencephalon and eye tissues in favour of diencephalic fates [57]. The expression of several secreted inhibitors able to bind either WNT molecules or their receptors is high in the anterior neural plate and AME. For instance, the anterior neural border of the zebrafish embryo is the source of a secreted WNT inhibitor, *tlc*, which is required for normal telencephalic development [60, 61]. However, the presence of these inhibitors is not the only way to ensure low levels of WNT signaling in the anterior neural plate.

**Fig. 3:**
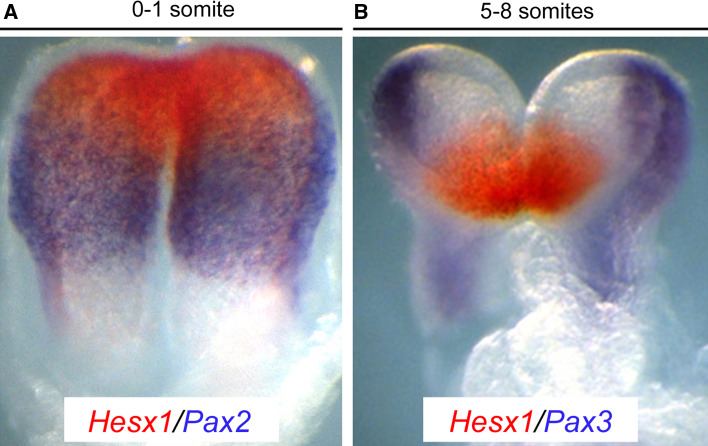
Subdivision of the neural plate in discreet gene expression domains. **a** At presomitic/early somite stages, in situ hybridization for *Hesx1* (*red*) and *Pax2* (*purple*), marking the anterior forebrain and posterior forebrain/midbrain precursors, respectively. **b** The anterior forebrain primordium express *Hesx1* (*red*), and *Pax3* (*purple*), delineates a posterior-lateral domain that gives rise to neural crest cells in an older embryo. Note that these domains do not overlap (adapted with permission from *Disease Models and Mechanisms* (Sajedi et al. (2008), *Disease Models and Mechanisms* 1 (4–5), 241–254)

Two early anterior neural markers, *Hesx1* and *Six3*, also contribute to inhibit the activation of the WNT pathway in a cell autonomous fashion (Fig. 3). *Six3* ^−*/*−^ mutant embryos show anterior forebrain defects with impaired telencephalic, eye, and hypothalamic development at 10.5 dpc, even though the anterior neural plate is initially specified at pre-somitic stages and expresses markers such as *Hesx1* [80, 109]. Failure to maintain anterior forebrain marker expression is associated with a rostral expansion of posterior markers such as *Pax3* and *Wnt1*. SIX3 can bind the *Wnt1* promoter and inhibit *Wnt1* expression in electroporated chick embryos, suggesting that SIX3 may repress the expression of *Wnt1* in anterior forebrain, preventing its posterior transformation. The simultaneous removal of *Six3* and *Wnt1* in *Six3* ^−*/*−^ *;Wnt1* ^−*/*−^ embryos improves diencephalic development, however, the telencephalon and eyes fail to develop as in the *Six3* ^−*/*−^ mutants [83]. This suggests that SIX3 may be required for anterior neural plate development independently of its ability to repress *Wnt1* expression [8, 22].

Very similar forebrain defects are observed in the *Hesx1* ^−*/*−^ mutants. Telencephalic vesicles and eyes are reduced or absent at early somite stages, but *Six3* expression is unaffected at neural plate stages [32, 95]. The lack of anterior forebrain is caused by a posterior transformation of the anterior neural plate, which was evidenced by the rostral expansion of posterior markers such as *Wnt1* and *Pax3*. In addition, and confirming the change of fate of the ANP, genetic fate mapping studies on *Hesx1* ^*Cre/*−^ *;R26* ^*YFP/*+^ (*Hesx1* null-mutants) and *Hesx1* ^*Cre/*+^ *;R26* ^*YFP/*+^ (normal) embryos have demonstrated that descendants of neural precursors initially fated to colonize the telencephalon and eyes end up populating posterior regions of the neural plate such as the thalamus, epithalamus, and pretectum [3]. In *Hesx1* ^−*/*−^ embryos, ectopic activation of the Wnt/β-catenin signaling pathway, evidenced by *Axin2* and *Sp5* expression, is detectable at early somite stages prior to the onset of *Wnt1* expression in the prospective midbrain/posterior forebrain region of the neural plate or *Fgf8* expression in the ANR. This suggests that the observed reduction of the *Fgf8* expression domain in the ANR and the expansion of the *Wnt1* expression domain are a consequence of the ectopic activation of the WNT/β-catenin pathway in the ANP. At the molecular level, gene profiling of isolated anterior forebrain precursors has demonstrated the ectopic activation of the WNT pathway in *Hesx1*-deficient embryos relative to controls [4]. Therefore, HESX1, as well as SIX3, may regulate the competence of the ANP making it unresponsive to the WNT molecules that are secreted at posterior levels (WNT1 and WNT3a). The regulation of cell competence, i.e., the ability of a cell to either respond or not to a given signal, is an important factor in cell fate specification that usually attracts less attention than the function of the inductive signals themselves [91, 133, 148]. For example, the expression domains of *Six3* and *Irx3* (a member of the Iroquois family of homeoproteins with a role in neural induction and patterning) have been proposed to confer distinct competence to inducing signals to specify anterior versus posterior neural plate identity [52, 53, 75, 120]. Analysis of the *Tcf7l1* (*Tcf3*) mutants supports the hypothesis that SIX3 and HESX1 may regulate cell competence within the anterior neural plate.

**Fig. 4:**
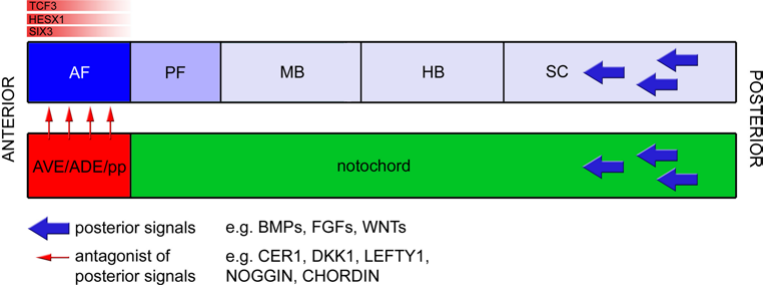
A host of signals act in concert to confer anterior forebrain identity. Signals such as *WNTs*, *FGFs*, and *BMPs* exert a posteriorizing action on the neural plate (*blue arrows*). Counteracting activation of the respective pathways in the anterior forebrain, secreted inhibitors such as Cerberus, *Lefty1*, *Dkk1*, Noggin, and Chordin (*red arrows*) are released by the underlying *AVE*, *ADE*, and prechordal plate. Within the prospective anterior forebrain, intrinsic factors such as HESX1, SIX3, and TCF3, aid in regulating the competence of neural tissue to not respond to posteriorizing signals, possibly by preventing the expression of target genes of these pathways, hence maintaining anterior forebrain identity. *AF* anterior forebrain, *PF* posterior forebrain, *MB* midbrain, *HB* hindbrain, *SC* spinal cord, *AVE* anterior visceral endoderm, *ADE* anterior definitive endoderm, *pp* prechordal plate

In mouse, *Tcf3*-deficient embryos undergo gastrulation, but exhibit variable degrees of defects, including primitive streak and axis duplications, supernumerary neural folds, and neural patterning defects involving expansion of midbrain at the expense of forebrain and hindbrain tissues [99]. At the molecular level, TCF3 acts as a repressor of WNT/β-catenin targets in *Xenopus* and zebrafish [18, 37, 69]. Genetic evidence in mouse has demonstrated that the expression of a mutant TCF3 protein lacking the β-catenin-interacting domain rescues the gastrulation and neural plate defects observed in the *Tcf3* ^−*/*−^ [160]. In addition, the conditional ablation of *Tcf3* within the anterior forebrain in *Hesx1* ^*Cre/*+^ *; Tcf3* ^*loxP/loxP*^ embryos leads to defects that are very similar if not identical to those observed in *Hesx1* ^−*/*−^ and *Six3* ^−*/*−^ mutants, such as small or absent telencephalon, hypothalamus, and eyes [4]. These data suggest that TCF3 is required for normal forebrain development as a transcriptional repressor of WNT/β-catenin targets. In addition, as *Tcf3*, *Hesx1*, and *Six3* are mostly co-expressed in the anterior neural plate at presomitic stages, it seems plausible that the three may co-operate at the protein level, ensuring that anterior forebrain precursors do not activate the expression of WNT targets, which could confer a posterior identity. Supporting this notion, there is a genetic interaction between *Hesx1* and *Six3* as well as between *Hesx1* and *Tcf3*, indicating that a minimum gene dose is required to maintain anterior neural plate identity [4, 50]. Although speculative, it is possible that *Rx*, another transcription factor may co-operate with *Hesx1*, *Six3*, and *Tcf3* in the maintenance of anterior identity [98]. Together, these data suggest that there is an additional level of control of the WNT pathway involving the expression of transcriptional repressors within the ANP that act to prevent an ectopic response to WNT signaling (Fig. 4). Further research will reveal the exact mechanisms by which these actions are mediated.

In conclusion, research over the last years has revealed the principles that regulate the development of the anterior forebrain at early stages of embryogenesis. There is a perfect interplay of signaling centers during normal morphogenesis of the embryo to ensure that the anterior forebrain is protected from caudalizing factors. This is initiated by the AVE, embellished by the ADE/AME, and reinforced by transcriptional repressors regulating cell competence within the anterior forebrain itself.
